# Land use conversion from peat swamp forest to oil palm agriculture greatly modifies microclimate and soil conditions

**DOI:** 10.7717/peerj.7656

**Published:** 2019-10-14

**Authors:** Subasini Anamulai, Ruzana Sanusi, Akbar Zubaid, Alex M. Lechner, Adham Ashton-Butt, Badrul Azhar

**Affiliations:** 1 Department of Forest Management, Faculty of Forestry, Universiti Putra Malaysia, Serdang, Selangor, Malaysia; 2 Institute of Tropical Forestry and Forest Product, Universiti Putra Malaysia, Serdang, Selangor, Malaysia; 3 Faculty of Science and Technology, National University of Malaysia, Bangi, Selangor, Malaysia; 4 School of Environmental and Geographical Sciences, University of Nottingham Malaysia, Semenyih, Selangor, Malaysia; 5 British Trust for Ornithology, The Nunnery, Thetford, United Kingdom; 6 Biodiversity Unit, Institute of Bioscience, Universiti Putra Malaysia, Serdang, Selangor, Malaysia

**Keywords:** Soil characteristics, Smallholding, Conservation, Plantation, Southeast Asia

## Abstract

Oil palm (*Elaeis guineensis*) agriculture is rapidly expanding and requires large areas of land in the tropics to meet the global demand for palm oil products. Land cover conversion of peat swamp forest to oil palm (large- and small-scale oil palm production) is likely to have negative impacts on microhabitat conditions. This study assessed the impact of peat swamp forest conversion to oil palm plantation on microclimate conditions and soil characteristics. The measurement of microclimate (air temperature, wind speed, light intensity and relative humidity) and soil characteristics (soil surface temperature, soil pH, soil moisture, and ground cover vegetation temperature) were compared at a peat swamp forest, smallholdings and a large-scale plantation. Results showed that the peat swamp forest was 1.5–2.3 °C cooler with significantly greater relative humidity, lower light intensities and wind speed compared to the smallholdings and large-scale plantations. Soil characteristics were also significantly different between the peat swamp forest and both types of oil palm plantations with lower soil pH, soil and ground cover vegetation surface temperatures and greater soil moisture in the peat swamp forest. These results suggest that peat swamp forests have greater ecosystem benefits compared to oil palm plantations with smallholdings agricultural approach as a promising management practice to improve microhabitat conditions. Our findings also justify the conservation of remaining peat swamp forest as it provides a refuge from harsh microclimatic conditions that characterize large plantations and smallholdings.

## Introduction

Tropical peat swamp forests are an important element of the world’s wetland ecosystems, as they serve as a dynamic bridge between land and water; they are an exchange zone for water flow and nutrient cycling. They have unique biophysical characteristics associated with their hydrology, soils and vegetation (Yule, 2010; Posa, Wijedasa & Corlett, 2011) and have a prominent global role as carbon sinks (Page, Rieley & Banks, 2011). Recent estimates of tropical peatland areas were 1,689,171 km^2^ (Gumbricht et al., 2017). Interestingly, of an overall 1.0% yearly forest cover decline in Southeast Asia, the highest deforestation rates were in peat swamp forests with an average annual rate of 2.2%, with the majority of forest being converted to plantations and secondary vegetation (Miettinen, Shi & Liew, 2011). If peatland deforestation continues at current rates (31,000 km^2^/year), Southeast Asian peat swamp forests are projected to disappear by around 2030 (Miettinen, Shi & Liew, 2012). The conversion of peat swamp forests will have major impacts on the local communities that depend on their ecosystem services, cause the extinction of endemic peat swamp forest species, increase global carbon emissions and climate change (Miettinen, Shi & Liew, 2012; Hawa et al., 2016; Sasidhran et al., 2016; Adila et al., 2017).

Expansions of agricultural activities, especially oil palm plantations are a major driver of deforestation (Tilman et al., 2001). An estimated over one million hectares of forest were cleared for oil palm agricultural activities between 1990 and 2005 in Malaysia with an increase from 2.4 to 4.2 million hectares over the period (Food and Agriculture Organization of the United Nations (FAO), 2016). The massive expansion of oil palm cultivation has led to concerns about impacts on natural habitats and biodiversity as well as greenhouse gas emissions. Changes in forest environments from human modification also alter forest climate (Hardwick et al., 2015) by effecting microclimates; where microclimate is defined in this paper as climate related to a specific area at the micro level (near the ground) that is dependent on local climatic characteristics and stand type (Camuffo, 2013; Aussenac, 2000). Long term cultivation and agricultural usage of peat swamp forest lead to a number of changes to microclimate, which include increased solar radiation interception, wind speed and air temperature and decreased moisture and vapor pressure (Luskin & Potts, 2011; Hardwick et al., 2015; Shuhada et al., 2017). Moreover, the modification of forests may also affect soil characteristics such as increased nutrient deficiency (oligotrophic) in bogs due to a lack of mineral input and leaching of organic compounds causing water to become extremely acidic (pH 4 or less) (Posa, Wijedasa & Corlett, 2011). In addition to changes in soil chemistry after land clearing, peat soil physical properties are negatively impacted in drained secondary peat swamp forests; which are recognized as important indicators of peat soil degradation (Firdaus, Seca & Ahmed, 2011).

Land use change may lead to disturbance of the ecosystem functions that negatively affect microclimate conditions and soil quality, influencing local, regional, and global climate processes and thus contribute to climate change on multiple levels. The implications of land use changes in microhabitat conditions on microclimate and soil characteristics are not very well studied. Moreover, differences in microclimate and soil characteristics between large-scale oil palm production using monoculture management system (single species and same age) vs. small-scale oil palm plantations smallholdings composed of mixed production (polyculture, crop species in oil palm plantation) (Azhar et al., 2015, 2017; Yahya et al., 2017) have never before been examined.

This study was conducted to determine the influence of peat swamp forest conversion on microclimate conditions and soil characteristics. We surveyed and compared between large- and small-scale oil palm production and peat swamp forest to assess the impact of land use conversion and production systems on microclimate conditions and soil characteristics. Our study gives an insight into the influence of habitat modification on abiotic factors on tropical peatlands in Southeast Asia; as even small changes in microclimate may have an impact on key ecological processes and biodiversity conservation (Larsen, 2012; Meyer, Sisk & Covington, 2001).

## Materials & Methods

### Study area

This study was conducted during dry season in the North Selangor Peat Swamp Forest (NSPSF) and nearby oil palm plantations on the West Coast of Peninsular Malaysia. NSPSF (N 3°40′26.56″, E 101°4′29.52″) is located in northwestern Selangor at 16 m above sea level. NSPSF covers an area of 73,593 ha, with 95% of logged peat swamp forest and 5% of dipterocarp forest (Azhar et al., 2011, 2013). In the study area the daily average maximum air temperature is 32 °C and the minimum is 23 °C, and the driest month is June with 139.4 mm of rain (Malaysia Meteorological Department, 2018).

**Figure 1:**
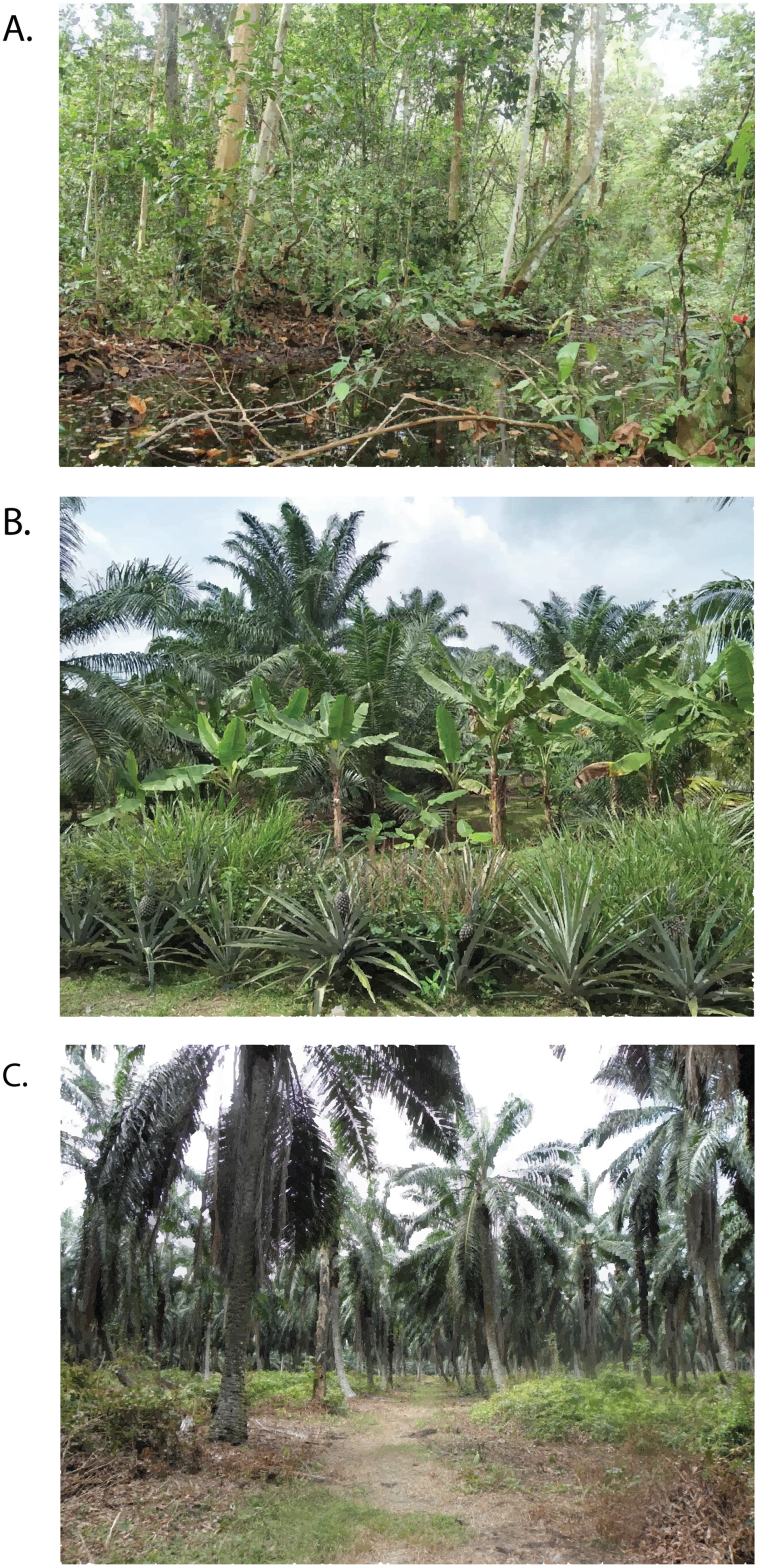
Peat swamp forest (A) is the original vegetation cover in the study area but large areas have been converted into oil palm smallholding (B) and large-scale plantations (C).

Three land uses were compared: (i) peat swamp forest, (ii) large scale plantation and (iii) smallholdings (Fig. 1). All these three land uses have distinct stand characteristics with different vegetation profiles and management regimes. Originally, both the large-scale plantation and smallholdings were peat swamp forests. The peat swamp forest site is a logged-over forest at which logging ceased 30 years ago (Azhar et al., 2011). The vegetation consists of a diverse range of peat swamp forest species that characterize the forest structure. The large-scale plantation is a monoculture of oil palm that has a minimum area of 2,000 ha operated by a corporation which use advanced machinery in their agricultural production systems (Azhar et al., 2011). The planting distances in oil palm plantations were standardized spacing of 7.8 m between rows and 9 m between pegs. The small scale plantation is distinctively different from the large scale plantation, as the smallholdings area covered less than 50 ha with semi-traditional cultivation areas that are managed by small-scale farmers who are less dependent on modern infrastructure and use mixed planting systems of monoculture intercropped with other economic crops (Azhar et al., 2015). However, all three land uses were closely located to ensure all the sites are in the peatland area and to diminish the geographical influence on this study. This data collection was verbally approved by the local land owners and by the organizations involved in writing.

### Study design

We used a systematic sampling design with random starting points to gather microclimate and soil data. We established 30 points, for a total of 90 in each land use type (e.g., peat swamp, plantation and smallholdings). The first sampling point was chosen randomly then all the other sampling points were at an equal distance apart (Morrison et al., 2008). A distance of 100 m was maintained between sampling points. All points were located at least 30 m from water-logged areas for logistics and safety reasons, as these areas are difficult to be traversed by walking. In addition, the sampling points were located at least 500 m from the edge, to minimize edge effects at all sites. For large scale plantation, all measurement points were within similar monoculture areas with similar stand ages in a systematic management system including the standardization in weeding and fertilizer applications while in the small scale plantation, the measurement points were made within the smallholding areas owned by multiple independent smallholders. This smallholding area is represented by oil palm stands that are characterized by variations in stand age and management system as they are self-managed, self-organized and self-financed with intercropping system that varies in the crop plants planted within the small scale oil palm plantations (Nagiah & Azmi, 2012; Azhar et al., 2015, 2017). The same sampling approach was also applied in the peat swamp forest. As the microclimate is highly related to localized daily climate conditions, the data collection was carried out for three cycles at the same sampling points to provide reliable microclimate regulation data thus minimizing the variations due to different daily local climate conditions (Wang et al., 2018). The data was collected between April 2016 until August 2016 (April 4–6, 2016; June 6–8, 2016; August 1–3, 2016) during the dry season.

### Measurement of microclimate and soil characteristics

Microclimate and soil characteristics were measured at all three land use types and for all 90 points. The microclimate parameters measured were air temperature, relative humidity, light intensity and wind speed. While the soil characteristics measured were soil pH, soil moisture, soil and ground cover vegetation surface temperature. All microclimate measurements were recorded during solar noon (12.00 pm–2.00 pm) when the sun is at its zenith (i.e., directly overhead) (Pereira, Nova & Galvani, 2003). Moreover, over the solar noon period the influence of vegetation on microclimate is expected to be at its greatest, such that differences amongst different land uses may be more evident (Sanusi et al., 2017). Measurements of air temperature, wind speed and relative humidity were made using the Skywatch ATMOS Thermo-Hygro-Anemometer (JDC Electronic SA, Yverdon-les-Bains, Switzerland) at 1.5 m above ground. All microclimate measurements were standardized by facing north. Wind speed was recorded every 2 s and averaged from 10 readings. Light intensity was measured with ISO-TECH 1332 digital Light Meter (RS Pro) at 1.5 m above ground and was measured every 30 s and averaged from 10 readings. For soil characteristics, soil moisture and pH were measured using a soil meter at a depth of 15 cm, recorded once a minute and averaged from three readings. Soil and ground vegetation surface temperatures were collected using a Fluke 59 MAX Infrared Thermometer (Fluke Corporation, Everett, Washington, D.C., USA) taken at 1.5 m above ground. For surface temperature, it was measured by holding the Infrared Thermometer perpendicular to the measured surface to ensure the surface area measured was consistent.

### Data analysis

We performed a one-way ANOVA with blocking to contrast the environmental variables (e.g., microclimate and soil characteristic parameters) between land use types. Different survey visits affect responses by creating variation and therefore are part of the error or noise aspect of the analysis. We treated them as nuisance factors where the effect of survey visits was included as the experimental block in this analysis. This provides a reliable representation of the microclimate condition of each land use and additionally minimizes the variation effects caused by the localized climate conditions. Tukey’s post hoc tests were done once significant differences were established to compare each land use group mean with every other group mean in a pairwise manner. The assumptions of the ANOVA concerning the residual or error terms (e.g., the normality, variance homogeneity, and independence) were examined before proceeding with the analysis. The data distribution was examined using Shapiro–Wilk test and Bartlett’s test for normality and homogeneity of variances, respectively. GenStat version 15 software was used for all analyses (VSNI; Hemel, Hempstead, UK). The raw data is available in the Supplemental File.

## Results

### Microclimate parameters

**Figure 2:**
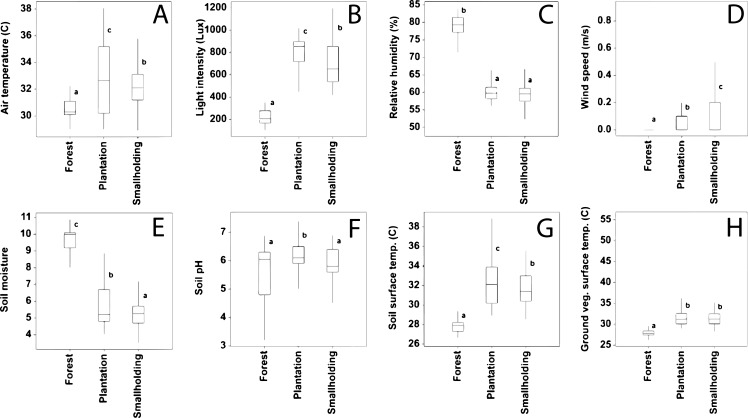
Boxplots of microclimate, soil and ground vegetation characteristics (A–H). The top and the bottom of the box are the first and third quartiles, and the central lined inside the box is the median (the second quartile). The whiskers represent the range of values; the minimum and maximum. Groups with the same letter are not detectably distinct and groups that are detectably distinct have different letters. If the groups have the same letter, there is no evidence of a difference for that pair.

There was a significant difference in air temperature between the peat swamp forest, smallholdings and large-scale plantation (variance ratio = 38.3; *P* < 0.001) (Fig. 2). The post hoc comparison showed that air temperature in the peat swamp forest (*x̅* = 30.5 °C; 95% CI [30.1–31.0 °C]) was significantly lower than in the smallholdings (*x̅* = 32.0 °C; 95% CI [31.5–32.4 °C]) and large-scale plantation (*x̅* = 32.8 °C; 95% CI [32.4–33.3 °C]). Moreover, the smallholding plantation had significantly lower air temperature compared to the large-scale plantation.

The difference in relative humidity between the peat swamp forest, smallholdings, and large-scale plantation was significant (variance ratio = 699.8; *P* < 0.001) (Fig. 2). Further statistical tests revealed that the relative humidity in the peat swamp forest (*x̅* = 78.7%; 95% CI [77.7–79.7%]) was significantly greater than the large-scale plantation (*x̅* = 60.8%; 95% CI [59.9–61.8%]), and smallholdings (*x̅* = 59.7%; 95% CI [58.8–60.7%]). The smallholdings did not significantly differ in relative humidity from the large-scale plantation.

There was a significant difference in light intensity (variance ratio = 348.7; *P* < 0.001) between the peat swamp forest, smallholdings and large-scale plantation (Fig. 2). Further post hoc analysis showed that light intensity in the large-scale plantation (*x̅* = 803.3 Lux; 95% CI [762.8–843.8 Lux]) was significantly higher compared to smallholdings (*x̅* = 700.7 Lux; 95% CI [660.2–741.2 Lux]) and the peat swamp forest (*x̅* = 214.1 Lux; 95% CI [173.6–254.6 Lux]). Smallholdings also significantly differed in light intensity to the peat swamp forest.

Finally, we found that wind speed differed significantly between the peat swamp forest, smallholdings, and large-scale plantation (variance ratio = 34.9; *P* < 0.001) (Fig. 2). Post hoc analysis indicated that wind speed in the smallholdings (*x̅* = 0.16444 m/s; 95% CI [0.13455–0.1943 m/s]) was significantly greater than those in the large-scale plantation (*x̅* = 0.07333 m/s; 95% CI [0.04344−0.1032 m/s]) and peat swamp forest (*x̅* = 0.01889 m/s; 95% CI [0.01100–0.0488 m/s]). In addition, the large-scale plantation significantly differed in wind speed to the smallholdings.

### Soil and ground vegetation characteristics

Soil moisture was significantly different between the three land uses (variance ratio = 343.7; *P* < 0.001) (Fig. 2). Post hoc comparison showed that soil moisture in the peat swamp forest (*x̅* = 9.5; 95% CI [9.2–9.8]) and smallholdings (*x̅* = 5.3; 95% CI [5.0–5.6]) significantly differed from the large-scale plantation (*x̅* = 5.8; 95% CI [5.5–6.1]). Significant difference in soil moisture was also detected between the peat swamp forest and smallholdings.

Our results showed significant differences in soil pH (variance ratio = 11.6; *P* < 0.001) (Fig. 2). Further post hoc analysis indicated that soil pH in the large-scale plantation (*x̅* = 6.2; 95% CI [6.0–6.4]) significantly differed from the smallholdings and peat swamp forest. However, the smallholdings (*x̅* = 5.8; 95% CI [5.6–6.0]) did not significantly differ from peat swamp forest (*x̅* = 5.64; 95% CI [5.4–5.8]).

Our results revealed significant differences in soil surface temperature (variance ratio = 199.4; *P* < 0.001) (Fig. 2). Post hoc comparison indicated that soil surface temperature in peat swamp forest (*x̅* = 27.8 °C; 95% CI [27.4–28.2 °C]) was significantly lower than in the smallholdings (*x̅* = 31.7 °C; 95% CI [31.3–32.1 °C]) and large-scale plantation (*x̅* = 32.3 °C; 95% CI [31.9–32.7 °C]). Smallholding significantly differed in soil surface temperature from large-scale plantation.

Lastly, there were significant differences in ground vegetation temperature between the peat swamp forest, smallholdings, and large-scale plantation (variance ratio = 94.3; *P* < 0.001) (Fig. 2). Further post hoc analysis showed that ground vegetation temperatures in the peat swamp forest (*x̅* = 27.9 °C; 95% CI [27.4–28.4 °C]) was significantly lower than the smallholdings (*x̅* = 31.4 °C; 95% CI [30.8–31.9 °C]) and large-scale plantation (*x̅* = 32.0 °C; 95% CI [31.4–32.5 °C]). Furthermore, the smallholdings did not significantly differ in ground vegetation temperature from the large-scale plantation.

## Discussion

Our findings suggest that a conversion to oil palm plantations from peat swamp forest creates hotter and drier microclimates as well as causing soil acidification. Similarly, the intensification of the microclimate conditions due to forest conversion to agriculture activities have been reported by other researchers in other regions in Malaysia and South-east Asia (Luskin & Potts, 2011; Hardwick et al., 2015; Shuhada et al., 2017). Conversions of peat swamp forest to commercial crops such as oil palm modifies microclimate and soil conditions, and are likely to have ecological implications on local biodiversity and at the landscape, and even global scale through contributing to climate change (Turner & Foster, 2009; Warren et al., 2017).

### Changes in peat land microclimates

This study showed that there were significant differences in the microclimatic parameters under the three different land covers: peat swamp forest, large-scale and smallholdings oil palm plantations. The air temperature, light intensity and wind speed in these three land covers were significantly different from one another. On the other hand, the smallholdings and large-scale oil palm plantation had significantly lower relative humidity compared to peat swamp forest, but no significant difference was detected between both plantation systems.

The large-scale plantation had the highest air temperature followed by the smallholdings and peat swamp forest. The results also showed that the peat swamp forest was 1.5 and 2.3 °C cooler compared to the smallholdings and large-scale plantation respectively. Similarly, Sabajo et al. (2017) also found that the conversion of forests to other land cover types such as oil palm, rubber and acacia plantation increased the land surface temperature in Jambi, Indonesia suggesting a local warming effect caused by the forest conversion. Moreover, the plantations were less humid compared to the peat swamp forest and hotter and drier than the natural forest regardless of its management system. Plantations will thus be a barrier to many forest species due to the harsher climatic conditions and likely reduce the dispersal ability of both flora and fauna across the landscape (Luskin & Potts, 2011).

Moreover, the light intensity and wind speed results were also in line with the above-mentioned results where the land conversion changed the amount of light penetration beneath the tree canopy and altered wind movement. Greater light intensity was recorded at the large scale and small holdings plantation compared to the peat swamp forest with differences of approximately 589 and 487 Lux respectively. This is largely due to reduced canopy coverage in the plantations which have shorter and more regular spaced oil palm trees with regular gaps between individual oil palm trees. According to Drescher et al. (2016), forested areas are characterized by a denser canopy and the conversion to oil palm plantation can significantly reduce plant diversity as well as increase canopy openness. Increase in canopy openness leads to greater light interception by and beneath the canopy (Ilo et al., 2014), which was observed in our study area as greater light intensities recorded in the plantations compared to the peat swamp forest.

At both local and global scale, wind regimes are a key factor that influences height-diameter allometries of tropical trees (Thomas, Martin & Mycroft, 2015). Conversely, stand structure plays an important role in influencing the wind movement; and in our study significantly greater wind speed was observed in plantation areas compared to the peat swamp forests. Trees alter wind movement by blocking wind from channeling (Flesch & Wilson, 1999; Wania et al., 2012; Sanusi et al., 2016) and stands with denser trees (i.e., dense peat swamp forest area) will theoretically have lower wind speeds due to obstructions caused by trees. These differences were observed in our study although wind speeds at all sites were relatively low ranging from 0.07 to 0.16 m/s. According to Komarudin, June & Meijide (2017), wind speeds will decline closer to the surface due to restrictions to air movement caused by the interaction between the surface and thus support what was observed in our study.

### Changes in soil characteristics

Soil pH, soil moisture, surface temperature of ground vegetation and soil surface temperature were influenced by the land conversion and there were significant differences between the peat swamp forest, large-scale oil palm plantations and smallholdings. Ground surface temperature varies depending on ground cover and the exposure from incoming solar radiation (Shashua-Bar, Pearlmutter & Erell, 2009). In plantations, greater openings in the canopy and a reduction in ground vegetation is likely to result in increased surface temperatures of the peat soil (Wösten et al., 2008).

The peat swamp forest had greater soil moisture than the oil palm plantations as both soil surface temperature and soil moisture were directly affecting each other. Soil moisture influences the surface temperatures through processes of evaporative control, thermal inertia and energy involved during evapotranspiration process of the bare soil and vegetated surfaces (Mallick, Bhattacharya & Patel, 2009). Moreover, according to Yates, Norton & Hobbs (2000), peat soil may lose moisture when burned or opened for agriculture purpose. According to Van Seters & Price (2001), peat swamp that has been harvested may not likely recover its ecosystem functions due to drainage and peat removal resulting in a lowering the water table which in turn leads to the inability to support *Spahagnum* moss renewal. Dense canopy in undisturbed peat swamp forest will protect and preserve soil moisture.

These findings are generally important for providing an understanding of below-canopy conditions when peat swamp forest is converted to oil palm agriculture and introduces extreme microclimate and changes the soil conditions in the area. This further justifies the need to conserve the peat swamp forest or use more sustainable agriculture systems to improve the microclimate and soil conditions at the landscape level.

### Large-scale plantation vs. smallholding

Microclimates are dramatically altered during plantation establishment in peat land areas (Luskin & Potts, 2011). A study by Drescher et al. (2016) suggest that land conversion of forest into rubber and oil palm leads to substantial losses in plant and animal diversity, reduced above- and below-ground carbon stocks and significantly affect microclimatic conditions. The deforestation of the peat swamp forest clearly causes an increase in sensible heat and uprising air temperature around the oil palm plantations.

Unfavorable impacts of land use changes from natural forest to agriculture was also clearly reported in this current study. Equally important, different agriculture managements systems also play a role in the modification of the surrounding microclimate and soil conditions. In the comparison between two oil palm plantations in our study, the large-scale plantation had significantly higher air temperature, light intensity, wind speed, soil pH, soil moisture, soil surface temperature and ground cover vegetation temperature compared to the smallholdings. This suggests that the polyculture agricultural system applied in smallholdings greatly improves the microclimatic and soil condition compared to the monoculture agriculture system in the large-scale plantation thus, highlights the different management of oil palm plantations has a great influence on the microclimatic and soil characteristics. This finding is in line with Böhnert et al. (2016) who also observed more severe microclimate conditions (i.e., air temperature and relative humidity) in monoculture rubber and oil palm plantations compared to the jungle rubber agroforestry (polyculture), thus further emphasize the potential of polyculture systems and in this case the smallholding plantations, in ameliorating the negative microclimatic changes associated with conversion from peat swamp forest.

In relation to this, differences in microclimatic and soil characteristics can be driven by canopy structure changes caused by natural or agricultural factors (i.e., management system) (Lugo & Gucinski, 2000). Large scale plantations apply monoculture systems while the smallholdings use mixed planting systems of monoculture and mainly intercropped with other economic crops. In smallholdings polyculture systems, multiple crops are integrated with oil palms, thus canopy cover is a mixture of different trees species creating vegetation heterogeneity and a heterogeneous canopy structure when compared to large-scale monocultures (Asmah et al., 2017). From the observation at these smallholding and large scale plantations, differences of the vegetation structure created from different planting designs and management between both plantations, greatly influence light penetration underneath tree canopies, thus affecting the microclimate and soil characteristics. Similarly, Ashraf et al. (2019) also highlighted that the alley-cropping system used in oil palm polyculture plantation improved the vegetation heterogeneity and microclimate regulation, however, it is also especially important to carefully selecting the crops when incorporating them with the oil palm trees as the selected crop characteristics and surrounding vegetation structural characteristics may influence the microclimate and soil conditions of an area. This emphasizes that polyculture agricultural systems used in smallholdings may serve as an alternative to sustainable agricultural practice and consequently contribute to overcoming global warming and biodiversity conservation challenges. Such an approach has the potential to be implemented in future agricultural landscape planning especially in Southeast Asia countries.

Apart from the polyculture management system, it is also important to note that there are other factors that can also influence the microclimate and soil conditions. Age stand may also contribute to the differences between the findings in the smallholdings and large scale plantations. For example, Ashraf et al. (2019) found that the monoculture oil palm plantation of different ages had different effects on microclimate conditions where a 15-year old oil palm showed lower solar radiation interception below canopy and higher relative humidity due to a more closed structure compared to a younger oil palm stand (7 year old).

On the other hand, natural peatland changes to agricultural oil palm also reduces the soil quality index as the changes affect the peat soil quality especially its peat depth, water-table depth and ash content (Nusantara et al., 2018). Change in water table depth decreases soil nutrients and affect the temperature and soil moisture of an area. Greater exposure to direct sunlight due to higher canopy openness increased the light intensity in the large-scale plantation compared to the smallholdings. This consequently resulted in significantly higher soil surface temperature and reduction of soil moisture in the large-scale plantation. This suggests that heterogeneous canopy structure such as observed in smallholdings may provide greater improvements in the surrounding microclimate and soil conditions. Moreover, as the components of vegetation in peatland has high interdependence with the peat substrate, disturbances on important elements of peatland such as water adequacy, canopy cover and leaf litter increase the fire risks that will negatively impact on ecosystems and consequently contributes to global climate change (Yule, 2010). It is important to note that all these factors demonstrate the need for careful planning of the future land use of the peatlands.

This current study compared large scale and small scale oil palm production, however, limitations which are associated with circumstances such as stand age, water table depth, management systems and oil palm density could not be controlled. In addition, future work could also include deeper soil temperature measurements which would be more informative since deep soil temperature better represents soil conditions over time and integrates the rates of microbial activity in the soil.

Despite the limitations of this study, it provides further direction for future land use planning strategies following small scale plantation operations that were observed to have greater vegetation heterogeneity as an alternative to large scale oil palm management systems, in order to improve microclimate and soil conditions. Most importantly, peat swamp forest conservation should be highlighted and supported as this natural peatland provides significantly greater ecosystem services in terms of microclimate and soil condition improvements compared to the smallholdings and large-scale plantations.

## Conclusions

Addressing oil palm expansion in the tropics is a great challenge for both local and national policy makers and conservation organizations worldwide. Zero conversion policy for peat swamp forests is imperative to ensure the continuity of this unique forest ecosystem. Particularly worrying, these environmental changes may also affect the biodiversity conservation efforts and jeopardize future soil viability for growing crops and/or forest regeneration. Future directions for land use planning strategies should consider the conservation of remaining peat swamp forest as it provides a refugia for biodiversity and to mitigate the detrimental impacts of climate change.

However, given the increasing global demand for palm oil, it is likely that peat swamp forests will be in danger from conversion to oil palm production in the near future. Our data suggests that land conversion from peat swamp forest to oil palm plantation greatly influences microclimate and soil characteristics. This research showed that different land uses lead to different microclimate and soil conditions with the peat swamp forest was up to 2.3 °C cooler compared to the smallholdings and large-scale plantations. Nevertheless, our study also highlights that the small-scale oil palm production (i.e., smallholding system) is likely to provide greater ecosystem services provision in terms of microclimate and soil characteristics compared to large-scale monoculture plantations and should be considered as one of the strategies to achieve sustainable oil palm production.

## Supplemental Information

10.7717/peerj.7656/supp-1 Supplemental Information 1 Raw data.

## Additional Information and Declarations

### Field Study Permissions

The following information was supplied relating to field study approvals (i.e., approving body and any reference numbers): Field collection was verbally approved by the local land owners and by the organizations involved in writing.

### Data Availability

The following information was supplied regarding data availability: The raw measurements are available in the Supplemental File.
